# Video support for prehospital stroke consultation: implications for system design and clinical implementation from prehospital simulations

**DOI:** 10.1186/s12911-024-02539-7

**Published:** 2024-05-29

**Authors:** Stefan Candefjord, Magnus Andersson Hagiwara, Bengt Arne Sjöqvist, Jan-Erik Karlsson, Annika Nordanstig, Lars Rosengren, Hanna Maurin Söderholm

**Affiliations:** 1 Department of Electrical Engineering, Chalmers University of Technology, Gothenburg, 412 96 Sweden; 2 Center for Prehospital Research, Faculty of Caring Science, Work Life and Social Welfare, University of Borås, Borås, 501 90 Sweden; 3 Department of Neurology, Sahlgrenska University Hospital, Gothenburg, Sweden; 4 Department of Clinical Neuroscience, Institute of Neuroscience and Physiology, Sahlgrenska Academy at University of Gothenburg, Gothenburg, Sweden; 5 Simulation Center West, Sahlgrenska University Hospital and University of Gothenburg, , Gothenburg, Sweden; 6 PICTA Prehospital Innovation Arena, Lindholmen Science Park, Gothenburg, Sweden

**Keywords:** Stroke, Prehospital care, Simulations, Video, Decision support, Digital health

## Abstract

**Background:**

Video consultations between hospital-based neurologists and Emergency Medical Services (EMS) have potential to increase precision of decisions regarding stroke patient assessment, management and transport. In this study we explored the use of real-time video streaming for neurologist–EMS consultation from the ambulance, using highly realistic full-scale prehospital simulations including role-play between on-scene EMS teams, simulated patients (actors), and neurologists specialized in stroke and reperfusion located at the remote regional stroke center.

**Methods:**

Video streams from three angles were used for collaborative assessment of stroke using the National Institutes of Health Stroke Scale (NIHSS) to assess symptoms affecting patient’s legs, arms, language, and facial expressions. The aim of the assessment was to determine appropriate management and transport destination based on the combination of geographical location and severity of stroke symptoms. Two realistic patient scenarios were created, with severe and moderate stroke symptoms, respectively. Each scenario was simulated using a neurologist acting as stroke patient and an ambulance team performing patient assessment. Four ambulance teams with two nurses each all performed both scenarios, for a total of eight cases. All scenarios were video recorded using handheld and fixed cameras. The audio from the video consultations was transcribed. Each team participated in a semi-structured interview, and neurologists and actors were also interviewed. Interviews were audio recorded and transcribed.

**Results:**

Analysis of video-recordings and post-interviews (*n* = 7) show a more thorough prehospital patient assessment, but longer total on-scene time, compared to a baseline scenario not using video consultation. Both ambulance nurses and neurologists deem that video consultation has potential to provide improved precision of assessment of stroke patients. Interviews verify the system design effectiveness and suggest minor modifications.

**Conclusions:**

The results indicate potential patient benefit based on a more effective assessment of the patient’s condition, which could lead to increased precision in decisions and more patients receiving optimal care. The findings outline requirements for pilot implementation and future clinical tests.

## Background

Stroke is one of the leading causes of mortality and disability worldwide, with over 100 million prevalent strokes and 12.2 million new cases in 2019, causing 6.55 million deaths and 143 million disability-adjusted life-years (DALYs) [1]. In the geographical context of this study (Sweden), stroke is the leading somatic illness with respect to cost and days spent in care facilities, with a societal lifetime cost per patient estimated to €68,000 [2]. Most stroke patients (87%) suffer from ischemic stroke (clot), whereas a minority (13%) suffer from nontraumatic intracerebral bleedings [3]. Out of the ischemic strokes around 10 to 20% are Large Vessel Occlusion (LVO) cases [3, 4], but this severe condition contributes disproportionately to ischemic stroke mortality (≈ 95%) and disability (≈ 60%) [3]. Stroke is a time critical condition, with direct correlation between *time to treatment* and *treatment outcomes.* Early recognition of stroke symptoms, e.g. at dispatch center, by Emergency Medical Services (EMS) clinicians (we use the profession-neutral term EMS clinicians to describe all kind of ambulance personnel since in Sweden various professions like registered nurses, emergency medical technicians, and physicians are involved) and through early notification to appropriate receiving facility, has positive effects on minimizing time to treatment delays [5, 6]. The EMS is an important link: EMS clinicians is the first point of access to care for the majority (> 70%) of patients with stroke symptoms. There are several challenges related to prehospital stroke assessment. Previous work indicate that EMS clinicians fail to identify 35% of stroke patients [7–9]. There are also risks that patients with stroke are misjudged and not transported to hospital by EMS at all [10].

One of the most important prehospital decisions, which will influence the *type of treatment* and *time to treatment*, is to determine the most appropriate care facility. Patients with LVO have highest chances of survival and good recovery when treated with thrombolysis (clot dissolving drug) followed by thrombectomy (mechanical removal of clot using a stent) compared to treatment with thrombolysis alone [11]. In Sweden, thrombectomy is provided by University Hospitals only. On the other hand, patients with clots in smaller vessels can be treated effectively with thrombolysis. In Sweden, thrombolysis can be performed in all hospitals with stroke units, which most often represent the facility with shortest transportation time. To minimize time to treatment for both types of patients, the challenge is to effectively differentiate the patients in need of thrombectomy versus thrombolysis. LVO patients exhibit more severe stroke symptoms, as measured by the National Institutes of Health Stroke Scale (NIHSS) [12]. The best cut-off value of the total NIHSS score to predict LVO has been reported to be 7 [13].

Today in Sweden, when suspecting a stroke, EMS clinicians usually call the emergency physician or a specialized nurse at the nearest local hospital to consult about the patient’s symptoms and to prepare the hospital for a possible coming thrombolysis case. The issue of thrombectomy and a secondary transport to regional stroke center is usually addressed during the thrombolysis procedure. As part of a regional initiative to improve and transform prehospital stroke care in Sweden, video consultation will be implemented with the aim to support a remote neurologist to perform stroke assessment using NIHSS, which has high accuracy for LVO detection but is challenging to use in prehospital settings since some signs can be difficult to investigate [14]. Previous work [15, 16] suggest that video is a reliable and feasible tool for remotely assessing stroke, with high agreement between remote and bedside assessments using NIHSS. Video consultation has also been successfully used to increase rate of thrombolysis in rural, underserved areas by setting up telemedicine networks between central and community hospitals in several countries, without adverse effects [17–20].

This study is part of a longer project called Video Support in the Prehospital Stroke Chain (ViPHS). It includes a step-wise development process following [21], including (1) process analysis and technical proposal; (2) realistic full-scale simulations; (3) limited operational pilot testing; (4) clinical implementation and benefit evaluation, in operational setting of a real-time video streaming solution between ambulances and a regional stroke center for prehospital stroke assessment. The aim of this study is to evaluate the first and second steps of the project focusing on the development and testing of video system design and implications for the prehospital work process using simulations.

## Methods

### Study design and setting

**Fig. 1:**
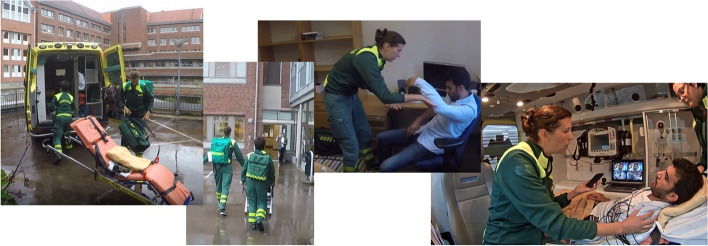
Simulation design illustrating EMS clinicians’ responding to a severe stroke that struck the patient (represented by an actor) at work

The present study had a mixed method design where simulations were used for data gathering. Simulations are one way to address research, design and evaluation challenges in healthcare. The prehospital context poses a number of research challenges, especially when it comes to observing specific procedures such as stroke assessment and care, e.g. aspects related to mobility/spanning several locations, and the unpredictability/difficulty to get access to the specific targeted patient cases or situations [22]. To explore the use and effects of video consultation on the prehospital stroke process, we used contextualized simulation scenarios [23] where real situations are recreated and reenacted by participants. The scenarios and environments were designed to be as realistic, engaging and immersive as possible [24]. To do this, we set up the simulations with a realistic workflow (Table 1) in real environments (e.g. a park bench outside, or an office at a university, a real ambulance); used real equipment (e.g. complete EMS bags, monitoring equipment, etc.); realistic stroke cases; realistic actors (neurologists with extensive experience of stroke patient assessment and stroke mimics); built in all information and access to information infrastructure as it would be provided normally (e.g. access to the real on-call neurology center, phone numbers and access to spouse/family member, dispatch information etc.) (Fig. 1).

**Table 1:** The phases and activities of the simulation. Guided by the description of the phases of ambulance missions in [25]. The simulation included all phases except handover. Numbers 1 and 2 indicate the two different scenarios (further info in Table 3)

*** Phase***	*** Activities during simulation***
**Receiving the call**	Five minutes of pre-driving time. The team assesses risks, plans the mission, organizes equipment, and gathers more information from the dispatch center.
**Arriving at the scene**	The team can assess the location, plan which equipment to bring in, and plan patient movement. 1) Colleague to patient meets and shows the way, or 2) passersby show where the patient is located.
**On-scene assessment and treatment**	1) The patient is seated on a chair in an office, or 2) on a bench outdoors. The team can begin patient assessment, make decisions regarding further examinations and treatments, determine the pace of care, and decide on the most appropriate transport to the ambulance.
**Transport decision and departure**	The team decides on transport to the ambulance, plans further examinations and treatments in the ambulance.
**En route assessment and treatment**	The team calls a neurologist via video, the team examines the patient together with the neurologist via video, the neurologist makes decisions about the destination of transport and treatment during transport. End of simulation.

The study was conducted in a region in the Southwest of Sweden, i.e. Region Västra Götaland. The physical location of the simulation was the yard and the first-floor offices of the University of Borås. The study included eight clinically active EMS clinicians (paired as four teams) from this specific region, and four remote consulting neurologists specialized in stroke and reperfusion located in the regional stroke center. When arriving, participants were introduced to the project and oral and written consent was obtained. They also got an introduction to the ambulance and the equipment they had available, and got some time on their own to rearrange and familiarize themselves with the EMS gear bag and the monitoring and communication equipment in the ambulance.

Each EMS team participated in two scenarios (Table 3), one where the patient exhibited symptoms of severe stroke (Scenario A), and one where stroke symptoms were moderate (Scenario B). In both scenarios, EMS clinicians were instructed to work as they normally do, the main difference being the video streaming to the remote regional neurologist, instead of contacting the nearest local hospital by telephone that is current standard procedure in the region. Participants took turn so that they performed one session each being the primary caregiver for the patient and consulted with the remote neurologist. Altogether eight unique neurologist–EMS clinician pairings were performed.

### The prehospital stroke assessment in the participant region

Prehospital stroke assessment follows local guidelines in Region Västra Götaland. When arriving on the scene of a suspected stroke victim, EMS clinicians are trained to follow a structured work procedure to assess the patient. Dispatch information sometimes include information regarding stroke suspicion, but sometimes not. On first patient contact, an initial assessment according to “Airway-Breathing-Circulation-Disability-Exposure (ABCDE)” [26] is performed. If a stroke is suspected, the EMS clinician should perform a stroke assessment based on a simplified version of NIHSS called (m)NIHSS (m for modified) [10]. This scale is a shorter version (eight items) of the complete NIHSS [27] (13 items) used at hospitals. It is used in Region Västra Götaland instead of e.g. the Face Arm Speech Test (FAST) that is the most common method in Swedish prehospital care. Assessments include e.g. motor skills of arms and legs, eye movements, understanding and language. Depending on the patient, situation and context, this is done on scene or in the ambulance. The simplified scale (m)NIHSS generates a score that reflects/indicates the severity of the stroke. According to regional stroke guidelines patients with suspected stroke should be transported to the nearest local hospital (for CT/MRI imaging and likely thrombolysis). However, within 45 min transportation time from regional stroke center, patients with scores ≥ 6 should initiate contact by phone with the responsible neurologist at the regional stroke center since there might be reason to bypass the local hospital and go directly to the regional stroke center for a decision regarding more advanced care (thrombolysis and thrombectomy). Patients with scores < 6 should be handled as all patients outside this zone and be transported to the nearest local hospital.

In the context of our study, there is one such center in the region (one regional stroke center per six local hospitals providing thrombolysis), i.e. Sahlgrenska University Hospital (Gothenburg, Sweden). It has a team of eight rotating neurologists staffing the on-call phone line that is open to the EMS ambulances within 45 min transportation time from regional stroke center. The (m)NIHSS score, in combination with information about symptom onset time provides the basis for a decision to transport the patient directly to the regional center for acute CT and potentially thrombolysis and thrombectomy. Alternatively, the patient is transported to the closest hospital for a possible thrombolysis and in case of a LVO confirmed by radiology, contact with the neurologist on-call for decision regarding secondary transport to the regional stroke center and thrombectomy.

### Video system design

Previous work on video applications in Sweden and Denmark for time critical remote assessment gathered through study visits, meetings and correspondence identified a number of aspects that influenced the system design. When designing the video system set-up, we drew on these ideas in combination with discussions in the project group. This group was a multidisciplinary team (*n* = 6) with researchers from neurology, prehospital care, engineering and information science (the authors). Together, the project group identified a set of initial, overall requirements (Table 2).

**Table 2:** Initial requirements identified by the multidisciplinary project team

**Requirement**	**Decision**	**Rationale**
Type of video set-up	Permanent; fixed cameras installed in the ambulance	Providing stable and standardized views and minimal technical operations for EMS clinicians
Number of views	3 (overview/fisheye, close-up, side-view)	Support for all the different types of NIHSS assessments (e.g. eye movements, arm/leg mobility, speech, etc.)
Remote camera control	Zoom, for face camera only Ability to switch views	Flexibility depending on what assessment that is performed, zoom function for face assessment
Awareness support/symmetric information	Provide EMS clinicians with a view of transmitted video streams	Common information space
Voice/audio	Mobile phone (on speaker)	To follow the normal EMS work procedure, facilitate audio recording, and for stability/reliability reasons during the test
Training	System use, (m)NIHSS, actors and equipment (EMS clinicians)	Participants

**Fig. 2:**
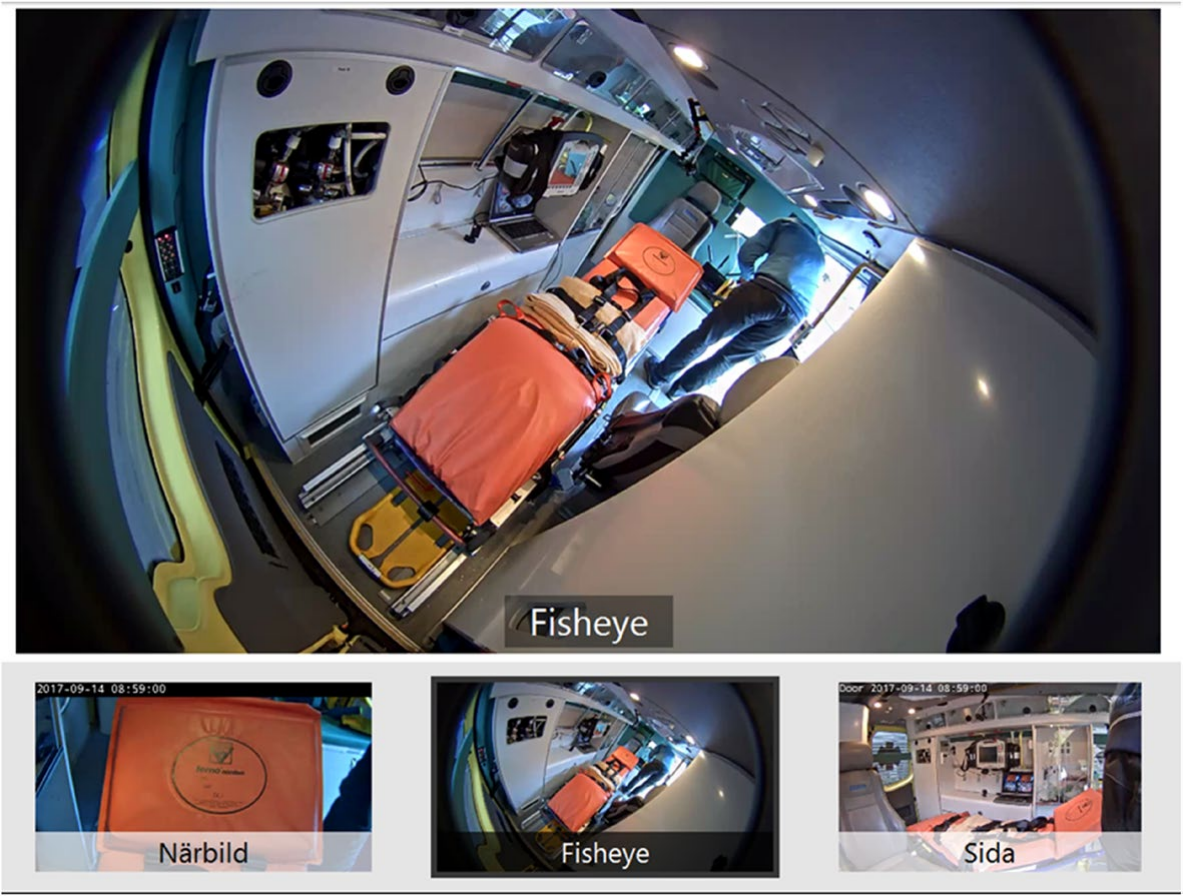
Remote view of the ambulance, including overview (“fisheye”, top and lower middle), close-up (“närbild”, lower left) and side-view (“sida”, lower right). Any of the three views could be selected as the main, enlarged view (top). The laptop placed to the left of the stretcher was used to provide the EMS clinicians with the possibility to see the images sent to the remote consulting neurologist

Based on the overall requirements, a working prototype was developed and installed as a permanent set-up in the patient compartment of an ambulance (Mercedes-Benz Sprinter 319 CDI, Germany). The patient compartment was equipped with three cameras, providing the remote physician with three different views of the patient, with the option to switch between these as main view (Fig. 2).

View 1 (overview) used a camera with so-called fisheye lens (Axis F1035-E Sensor Unit, Axis Communications AB, Lund, Sweden). The overview camera provided 1080p (1920 × 1080 pixels) resolution and 194° horizontal field of view. It was mounted at the back of the patient compartment. Its purpose was to capture as much of the interior of the ambulance as possible, and especially to capture the patient’s leg movements during stroke assessment. View 2 (side view), used a wide-angle camera (Axis A8105-E Network Video Door Station) mounted at the wall opposite to the patient. This camera provided 1080p resolution and 180° horizontal field of view, with purpose to capture the patient’s upper body from the side and especially arm movements during stroke assessment, as well as the EMS clinicians. It also included a microphone to record the conversation; however, mobile phone was used as audio source for the consultation in this study following standard clinical routine. The advantage of telephone as an audio source is that if video communication were to shut down or become unstable, EMS clinicians and neurologists would continue to be in contact. View 3 (close-up) was provided using a varifocal lens (Axis F1015 Sensor Unit). It provided between a 52° and 97° horizontal field of view, and was adjusted to maximum zoom at 52°. Its purpose was to capture a close-up image of the patient’s face for assessing facial expressions, eye movements and pupil size and dynamic behavior. A laptop was set up in the ambulance (Fig. 2) that supplied the video streams in four-split screen mode. The rationale for this was to give the EMS clinicians the possibility to see the video streams sent to the remote supporting neurologist, to facilitate the communication and assure that the neurologist was provided appropriate views for assessing the patient.

The F1015 and F1035 sensor units were connected to a video server (Axis F44 Dual Audio Input Main Unit). The software Axis Companion (Axis Communications) was used to connect the stroke neurologist team at the regional stroke center with the stationary ambulance (approximately 60 km away). The neurologists used a laptop to view the video streams. The software provided a possibility to digitally zoom into a selected region/point in the main view. The video system was connected via Ethernet, providing stable and fast Internet connection. The videos were recorded using the Axis F44 unit and SD memory cards. The video format was H.264.

### Data collection

We developed two scenarios, one with a patient with a more severe stroke, and one with a patient with moderate stroke symptoms (Table 3). The scenarios were based on real anonymized patient cases, with minor modifications to better suit the simulation setup. They were developed by two neurologists in the research team (authors JEK and LR).

**Table 3:** Description of the two stroke scenarios. Patients were represented by actors

	Scenario A: Johan, 54 years old	Scenario B: Ilena, 70 years old
		
Background	Previous diseases are hypertension, hyperlipidemia and type 2 diabetes. Works in his office when he suddenly falls ill with dizziness, nausea and visual disturbances with double vision.	Previous diseases are COPD, hypertension, hyperlipidemia, operated aortic aneurysm 2011, impaired kidney function, treated depression. Is out walking when she feels weird. Sits down and has difficulties to move left arm and leg. Calls a friend who calls an ambulance.
Context	Normal office space. The patient is sitting in an office chair. Is awake but generally affected.	Sitting on a bench outside. Leaning to the left. No other people in place.
Symptoms	Weak left side. Vague and difficult-to-understand speech. Normal vital parameters and ECG.	Can support on the right leg when moving to the ambulance stretcher. Leaning to the left on the stretcher. Slightly high blood pressure and serum glucose. Otherwise normal parameters.
	Severe stroke (NIHSS = 21)	Moderate stroke (NIHSS = 10)

The two scenarios were video recorded using one handheld and three fixed GoPro-cameras. The parts of the scenarios that took place inside of the ambulance were recorded via the three video cameras used for the consultation (described above). Audio from the video-consultations was transcribed (authors HMS, SC and MAH). After the two scenarios were completed, each team participated in a semi-structured interview (similar to [28]) regarding their perspectives on using the video-system, its design and technical set-up, and what they thought about communicating with the neurologist with respect to the different NIHSS assessments and their mutual understanding. We also asked about feedback on the simulation design and realism. Individual interviews with participating neurologists were conducted at their workplace at the hospital after all simulations were completed. All interviews were audio recorded and later transcribed (author HMS).

### Data analysis

The video recordings were analyzed with respect to *time, patient assessments* and *decisions made*, and analyzed using descriptive statistics. They were time-stamped to determine time spent on-scene with the patient, in the ambulance, duration of the video consultation and total on-scene time (from arrival to transport decision). To evaluate EMS clinicians’ on-scene patient assessment, we used an instrument for competence assessment by Tavares [29] and the translated and cultural adapted instrument by Bremer et al. [30]. To this point, there are no clinical studies investigating prehospital patient assessment in this level of detail, hence we provide data collected in a previous study [31] on prehospital stroke assessment, using the same assessment protocol and type of simulations as we do here. Mann-Whitney U tests were used to determine differences between the historical baseline group and the group participating in the present study.

Transcripts of team interviews and of the interviews with neurologists were analyzed using open coding, identifying broad initial categories that were later refined into themes and sub-themes pertaining to e.g. validity of the simulation and audio/video components of the system design.

### Ethical considerations

The present study is not within the boundaries of the Ethical Review Act 2003:460 that regulates all types of research involving humans in Sweden. The research was however conducted in accordance with the requirements of the Declaration of Helsinki [32]. This was accomplished through informed consent of all participants. The participant was informed that they participated voluntarily and that they could cancel their participation at any time and without justification and all data concerning their person should be deleted. All participants consented orally and in writing to all results including images being published. All video and interview data are stored at the university on password-protected servers.

## Results

### Face validity

All EMS clinicians (*n* = 8) expressed high satisfaction with their participation in the study, both with respect to be involved in and contribute to the development of the video consultation procedure, and to the realism of the simulation scenarios. In particular, they appreciated the acting skills of the two neurologists that played the roles of the patients, illustrated by comments such as the following:

*“This has been really really good… This is among the best experiences I’ve had when it comes to acting ability (---) Especially the motor activity and the way that they respond and looks at you (…) like when she was supposed to act as an elderly person, not that she was slow but there were a certain slowness.(---) He could really have fooled us! When it all had ended and he started to relax and get back to his real self, I just “God he is well! I mean, he’s not ill!” (---) You really got into it and thought that ‘now I’m working’. And also that you are in an ambulance. The environment is the right one.” (team 4)*.

*“When we’re out there, we know the variations there are out there, and what we encountered here, that is what we encounter in real life, so that made it even better. They [the patient actors] knew what an ill patient and a less ill patient looks like” (team 2)*.

All teams expressed that they had sufficient introduction to the simulation and enough time to familiarize themselves with the ambulance environment and video equipment.

### Patient assessment

To understand how video consultation may affect the prehospital stroke work process, we analysed patient assessment activities and durations in the simulated scenarios and compared these to a historical baseline. Overall, EMS clinicians in this study performed a more detailed patient assessment on scene, in closer agreement with recommended number of enquiries, questions and parameters according to regional stroke guidelines, as compared to baseline (Table 4).

**Table 4:** EMS clinicians’ patient assessment evaluation and comparison (the number of conducted enquiries, questions to the patient, and measurement of vital parameters)

**Patient assessment items**	**Baseline** median (range) (1 stroke simulation case, 11 EMS teams)	**Video** median (range) (2 stroke simulation cases, 4 EMS teams)	***P***
First survey (recommended enquiries *n* = 5)	3.0 (1–5)	4.5 (3–5)	0.03*
History gathering (recommended questions *n* = 14)	6.0 (4–8)	9.5 (7–11)	< 0.01*
STROKE assessment (recommended enquiries *n* = 13)	7.0 (5–10)	12.5 (11–13)	< 0.01*
Vital parameters (recommended parameters *n* = 7)	6.0 (4–6)	5.5 (4–6)	0.96

*Significant difference *p* < 0.05 using a Mann-Whitney U test

Video recordings also revealed three common approaches to EMS clinicians stroke assessment. The teams:

1. performed an incomplete (m)NIHSS on scene before the video consultation.
2. performed a complete (m)NIHSS on scene and then again repeated the assessments together with the remote neurologist during NIHSS assessment.
3. performed a complete (m)NIHSS assessment and reported the outcome when initiating the video consultation.

### On scene time

We measured the time EMS teams spent on-scene with the patient, in the ambulance, and total on-scene time before departure. When consulting via video, the median total on-scene time for all sessions was approximately 22 min (Table 5):

**Table 5:** Comparison of patient treatment and assessment median times for baseline and video consultation

**Time (mm:ss)**	**Baseline (MD)** **(1 stroke simulation case, 11 EMS teams)**	**Video (MD)** **(2 stroke simulation cases, 4 EMS teams)**	***p***
Time on scene *Range*	10:23 (04:09–15:23)	05:09 (03:16–09:01)	0.02*
Time in ambulance before departure *Range*	04:18 (02:29–09:21)	16:32 (12:34–22:30)	< 0.01*
Total on scene time *Range*	15:50 (08:01–22:13)	21:53 (18:36–26:30)	0.02*

*Significant difference *p* < 0.05 using a Mann-Whitney U test

*MD* median

Compared to baseline, EMS teams in this study were quicker to move the patient into the ambulance. The overall time inside the ambulance when using video was about 12 min longer. This is not surprising, considering that this is where the video consultation took place. In all, using video as set up in this study added approximately 6 min to the total on scene time (from arrival to departure). Note that these times are solely from simulation scenarios with participants with no prior experience of using video, and may only be indicative of trends in a real clinical setting.

### Transport decisions

For the patient with severe stroke symptoms (Case A), direct transport decisions were taken in all four simulations, no matter if the stroke took place in a rural location or close to a local hospital. For the patient with moderate stroke symptoms (Case B), two decisions were made to do direct transport and two to transport to a local hospital, with equal distribution between rural location and close to a local hospital (Table 6).

**Table 6:** Transportation decisions

**Decision**	Case A: Severe stroke symptoms		Case B: Moderate stroke symptoms		**Total sum**
	* Rural location*	* Close to local hospital*	* Rural location*	* Close to local hospital*	
Direct transport to regional stroke center	2	2	1	1	6
Transport to local hospital	0	0	1	1	2

### System design

**Fig. 3:**
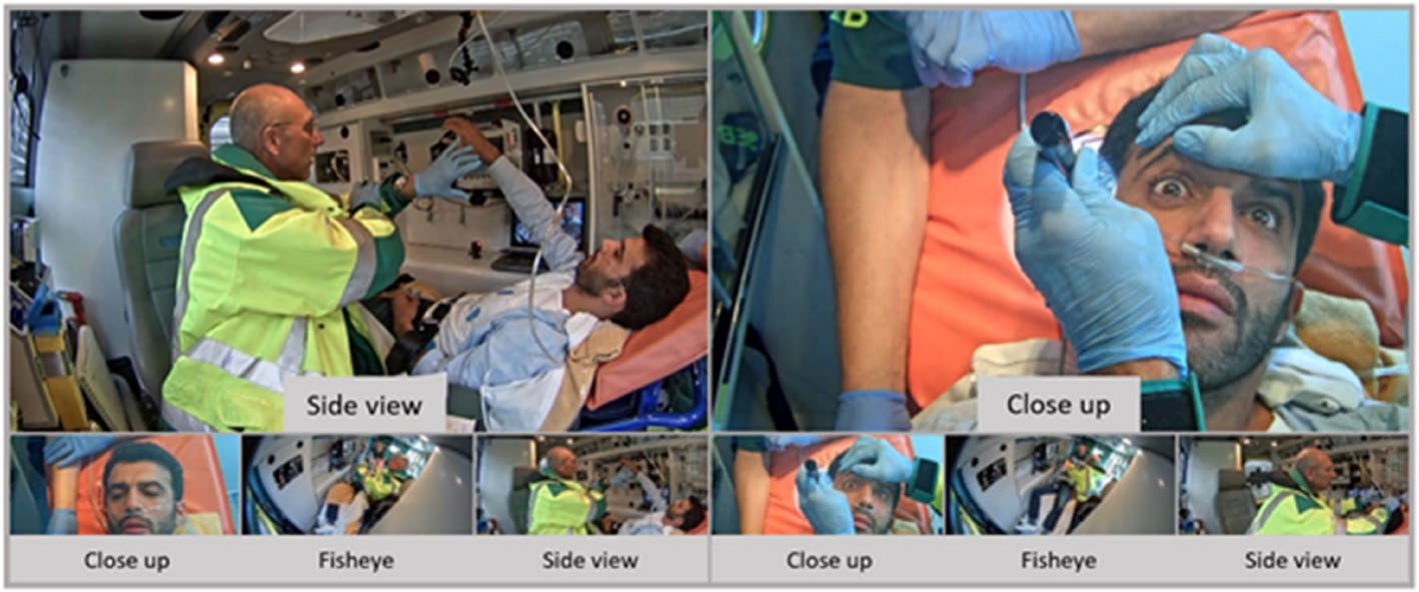
Neurologists view during consultation, views cover both EMS clinician and patient. Patient was represented by an actor

Overall, participants were satisfied with the technical set-up. None of the EMS clinicians said they were disturbed by the cameras, or thought that much about being filmed. Neurologists appreciated the three views that were provided, although reported that they did not switch much between views, but most often used the side view and sometimes the close-up. The resolution of the close-up image was perceived as sufficiently high to allow assessment of pupil movements by using the digital zoom function (Fig. 3).

Regarding the usefulness of the screen in the ambulance of the transmitted video stream, participants reported different experiences.

*“Yes, sometimes you looked at it to see just the eye movement and stuff like that, the patient is lying with his face up against the camera or is it on the side a little close, or do I cover something now when I sit here, when you are still in front and stuff. It gave a good idea, what was seen and not” *(team 3).

There were some suggestions on physically moving the screen further away from the patient to avoid agitated patients hitting it, and to avoid exposure to various body fluids or dirt from outside. When asked about if they would have liked to be able to see the physician, none of the EMS clinicians felt that it was necessary. It could however be helpful when the physician had to explain how to perform a certain NIHSS item or additional assessments that the EMS clinicians are unaware/lack knowledge of.

Participants were least satisfied with the design of the audio component, both with respect to audio quality and format (handheld speaker phone). They reported difficulties to hear the neurologist, and the neurologists found it difficult to hear the patient. Furthermore, it was impractical and sometimes impossible to hold the phone while interacting with and performing assessments on the patient. Often, the secondary EMS clinician in the team assisted by holding the phone. Several suggested using a headset, or at least the option to switch from speaker mode to private conversation with the neurologist. Several different reasons were mentioned to why and why not the patient should be able to hear or take part in the conversation:

*“It was as we talked about after my assignment that some patients are like that and so, they do not want to be sick, so many times, she said (neurologist) that “Yes, but do the assessment with both fingers” so we see if she sees both fields of view and then the patient heard it and then she could say “Yes, but it is on both sides” even though it really is not.”* (team 4).

*“If the patient, or the doctor himself wants to talk to the patient, then you can of course turn on the speaker, but otherwise I would have really liked to have it in my ear.”* (team 4).

This indicates that there should be an option to be flexible with how audio is distributed depending on patient and situation.

### Requirements for pilot implementation

Based on the results from the simulations, the project team identified several requirements that were deemed important before pilot implementation (Table 7).

**Table 7:** Requirements for future pilot implementation

**Requirement type**	**What**	**Comment (based on our study results)**
Set-up/system design	Permanent; fixed cameras installed in the ambulance	Video coverage and quality was good, enabling neurologists to assess the patients’ symptoms remotely. Per suggestion, we will try a mobile set-up in a separate, subsequent project.
Number of views	3 (overview/fisheye, close-up, side-view)	Worked well according to neurologists. No obstruction of view by EMS clinicians was noted or reported.
Remote camera capabilities	Zoom, for face camera only	Digital zoom was deemed sufficient to allow assessment of pupil movements. Optical zoom would enable higher resolution at extreme close-up images.
Awareness support/symmetry	1) Provide EMS clinician with a view of transmitted video stream 2) Provide EMS clinician with physician thumbnail/name on screen and or video	1) If possible but probably not crucial other than for error detection. 2) Seeing the name of the physician on the screen would be useful. Seeing the physician on video is not necessary, other than for easier instruction on how to perform some of the NIHSS assessments (could also be addressed through training).
Voice/audio	Mobile phone (on speaker)	Integrated audio/voice system No hand-held unit Option to switch between speaker mode and headphones
Work process	Prehospital process guideline and (m)NIHSS	There needs to be a consensus on how (m)NIHSS/NIHSS should be used: what assessments should be done before the consultation and what should be reported and/or repeated.
Training and documentation	(m)NIHSS form NIHSS form	In order to establish a common point of reference: provide EMS clinicians with the complete NIHSS form that the neurologists use, in addition to the (m)NIHSS form in the ambulance. Check so that the items in the two guidelines correspond and use the same terminology

## Discussion

This study developed and implemented an ambulance-based video system and evaluated its potential clinical benefit for collaborative stroke patient assessment by EMS clinicians and remote neurologists specialized in stroke and reperfusion. To our knowledge there are few evaluations of such systems published in the scientific literature [33, 34]. The results were overall promising and indicate a potential clinical benefit to improve acute treatment of stroke patients, mainly by increasing the precision of transport decisions. Ambulance nurses and neurologists were satisfied with system performance and deemed that video consultation has potential to improve precision of stroke symptom assessment. The collaborative assessment worked well and was experienced as helpful for deciding whether to transport the patient directly to the regional stroke center or to the closest hospital.

The simulations were based on previous research [31] and were considered realistic, as found from the interviews with all participating clinicians, who expressed that the environment was familiar and the actors captured stroke patients’ symptoms well. We believe that simulation was an effective tool in safely evaluating the potential of video consultation, to attain increased understanding of how the system can be used effectively for stroke assessment, before commencing with clinical implementation.

The total time for patient assessment increased as compared to the historical baseline, by on average 6 min. It is expected that a more thorough assessment will increase the on-scene time needed before transportation. However, we consider the added time by using video for collaborative assessment in relation to the potential for overall shorter time to the right treatment to be potentially clinically beneficial. The reason is that taking an inappropriate transportation decision can produce substantial delays to treatment, typically in the order of hours due to need of transferring patients from the local hospital to the regional stroke center [4]. Many patients in need of thrombectomy may not receive that treatment at all since the time window for effective thrombectomy treatment is missed. Fewer than 2% of patients with ischemic stroke were reported to have been treated with thrombectomy in Europe 2019 although 11–20% of these patients are estimated to have LVO [4].

The total assessment time when using video and remote consultation can likely be decreased by streamlining the procedure. In the present study some assessments, e.g. NIHSS items, were repeated seemingly unnecessarily. No extensive training preceded the simulations performed here, nor was any complete recommended protocol developed that could guide what is needed to do before calling the neurologist. Instead, the aim was to test video consultation in a working scenario closely resembling the EMS clinicians’ ordinary work situations. The repeated assessments made the total assessment time prolonged. Based on this study it will be of importance to develop a protocol for the prehospital work process in connection to stroke symptoms and video consultation. For example, guide the EMS clinicians to abort their (m)NIHSS assessment on site when 6 points have been achieved and instead transport the patient to the ambulance for further NIHSS assessment via video. Another recommendation is to describe the consultation itself in a protocol, for example that EMS clinicians report the patient initially and ask “how do we proceed” and then the neurologist takes over the assessment. Furthermore, transportation times to the regional stroke center and other nearby hospitals could be automatically calculated and when the regional stroke center is within close reach there may not be a need for video consultation. Other LVO detection tools including machine learning based approaches should also be considered in the future [14, 35, 36].

The main limitation in the present study was the simulated environment. There is, of course, a risk that the video consultations and patient assessment would have been significantly different in a real ambulance mission. Another shortcoming is that the study used a retrospective control group from another simulation experiment with stroke cases. This control group had similar patient cases but not exactly the same. The experiment only filmed the work of EMS clinicians. The neurologists who sat at a distance were not filmed. Thus, there is a lack of knowledge about how the neurologists actually worked in the system. Furthermore, the number of EMS teams was relatively low, so the statistical results are merely an indication of possible trends.

In future work video consultation will be pilot tested in a limited clinical setting. Practical issues like bandwidth limitations need to be handled. Clinical benefit will be evaluated by examining times for thrombolysis and thrombectomy treatment, respectively. Other valuable evaluations from a pilot test are to improve the work process in connection with video consultation via interviews with EMS clinicians and neurologists.

Further ahead we believe that video may be developed into being a natural part of the EMS toolset, and also be a support for other patient groups [37]. In particular, time-critical incidents with specialized treatment options may benefit from video consultation with experts. For example, trauma care is limited by high rates of undertriage [38, 39], which indicates challenges with patient assessment that potentially could be aided by video consultation in conjunction with other tools for improved decision support [40]. In addition to human experts, computer vision driven by artificial intelligence (AI) progress could possibly assist in patient assessment. As one example, standardized assessments such as NIHSS items that describe leg and arm movements, and facial expressions may be quantified and evaluated by AI [41]. Such computerized processing conveys ethical and privacy issues that need to be resolved. How video data should be handled needs to be decided, e.g. whether the data should be recorded and saved in the Electronic Patient Record (EPR) or only streamed live without saving, which has implications on patient and EMS clinician integrity as well as loss of potentially valuable clinical data.

## Conclusions

This study developed and evaluated a video setup for collaborative prehospital stroke assessment with remote neurologists. Both ambulance nurses and neurologists are content with the technical setup and deem that video consultation has potential to provide improved precision of stroke symptom assessment. This could aid in identification of patients that can benefit from direct transportation to a regional stroke center, to lower the time from call to definitive treatment and increase the proportion of patients that can be offered thrombectomy. In a following phase the technology will be integrated into the regional IT and video platform and evaluated in real patient cases.

## Data Availability

The datasets generated and/or analysed during the current study are not publicly available but are available from the corresponding author on reasonable request.

## Abbreviations

ED: Emergency Department
EMS: Emergency Medical Services
LVO: Large Vessel Occlusion
(m)NIHSS: modified National Institutes of Health Stroke Scale [10]
NIHSS: National Institutes of Health Stroke Scale
ViPHS: Video Support in the Prehospital Stroke Chain
